# Activation of PPAR-γ prevents TERT-mediated pulmonary vascular remodeling in MCT-induced pulmonary hypertension

**DOI:** 10.1016/j.heliyon.2023.e14173

**Published:** 2023-03-04

**Authors:** Tafseel Hussain, Limin Chai, Yan Wang, Qianqian Zhang, Jian Wang, Wenhua Shi, Qingting Wang, Manxiang Li, Xinming Xie

**Affiliations:** a Department of Respiratory and Critical Care Medicine, The First Affiliated Hospital of Xi'an Jiaotong University, Xi'an, Shaanxi, PR China; b Department of Respiratory and Critical Care Medicine, The Second Affiliated Hospital of Xi'an Jiaotong University, Xi'an, Shaanxi, PR China

**Keywords:** Pulmonary vascular remodeling, Peroxisome proliferator-activated receptor γ, Pulmonary artery hypertension, Telomerase, Telomerase reverse transcriptase, Akt/c-Myc

## Abstract

**Background:**

It has been demonstrated that elevated telomerase reverse transcriptase (TERT) expression or activity is implicated in pulmonary hypertension (PH). In addition, activation of peroxisome-proliferator-activated receptor γ (PPAR-γ) has been found to prevent PH progression. However, the molecular mechanism responsible for the protective effect of PPAR-γ activation on TERT expression in the pathogenesis of PH remains unknown. This study was performed to address these issues.

**Methods:**

Intraperitoneal injection of monocrotaline (MCT) was used to establish PH. BIBR1532 was applied to inhibit the activity of telomerase. The right ventricular systolic pressure (RVSP) and histological analysis were used to detect the development of PH. The protein levels of p-Akt, t-Akt, c-Myc and TERT were determined by western blotting. Pharmacological inhibition of TERT by BIBR1532 effectively suppressed RVSP, RVHI and the WT% in MCT-induced PH rats.

**Results:**

Pharmacological inhibition of Akt/c-Myc pathway by LY294002 diminished TERT upregulation, RVSP, RVHI and WT% in MCT-PH rats. Activation of PPAR-γ by pioglitazone inhibited p-Akt and c-Myc expressions and further downregulated TERT, thus to reduced RVSP, RVHI and WT% in MCT-treated PH rats.

**Conclusions:**

In conclusion, TERT upregulation contributes to PH development in MCT-treated rats. Activation of PPAR-γ prevents pulmonary arterial remodeling through Akt/c-Myc/TERT axis suppression.

## Graphical abstract

**Figure:**
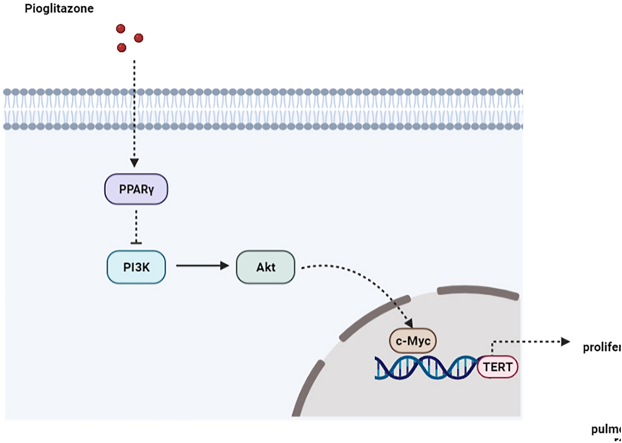

## 1 Introduction

Pulmonary arterial hypertension is a severe disease characterized by abnormal structure and dysfunction of pulmonary blood vessels caused by multiple factors, leading to the sustained increases in pulmonary vascular resistance and pulmonary arterial pressure, and eventually right heart failure. Currently, the main pathological mechanisms underlying PH compromise persistent pulmonary vasoconstriction, vascular remodeling and thrombosis in situ [1,2]. Pulmonary vascular remodeling has been considered as the major structural alteration of PH, in which aberrant proliferation of pulmonary arterial smooth muscle cells (PASMCs) plays the key role in this process. Therefore, elucidating the molecular mechanisms responsible for PASMCs proliferation in pulmonary vascular remodeling is essential for identifying novel treatments of PH.

Telomerase reverse transcriptase (TERT) encodes the catalytic subunit of telomerase to maintain telomerase activation [3,4]. Telomerase reactivation is a prerequisite for cell immortalization, and telomerase over expression exists in more than 85% of human tumours [5,6]. Recent studies have suggested that TERT is upregulated and activated in PH patients and a rat model of PH [7,8], and that TERT regulates cyclin D1 and G1/S phase transitions to promote cell proliferation [9,10]. Knockdown of TERT in an animal model of PH effectively alleviated PASMCs proliferation and pulmonary vascular remodeling [11]. Recently, we have reported that PDGF promotes PASMC proliferation and migration through the Akt/c-MYC/TERT axis in vitro. Furthermore, activation of PPAR-γ with pioglitazone suppressed PDGF-induced TERT expression and telomerase activation, leading to inhibition of PASMC proliferation and migration [12]. However, the underlying mechanisms of how individual targets in the above pathways mediate PASMC proliferation and pulmonary vascular remodeling in vivo are not fully understood.

The PI3K/Akt signaling pathway is an intracellular signal transduction pathway that is involved in many cell process, including cell metabolism, proliferation, survival, and angiogenesis [1,2,13,14]. Abnormal activation of the PI3K/AKT pathway has been found to promote PASMCs proliferation in PH [15]. Recent studies have demonstrated that activation of PI3K/Akt mediates TERT upregulation to promote tumour cell proliferation [16]. Besides, inhibition of PI3K/Akt pathway suppresses lung cancer proliferation via blockage of TERT expression [17]. These studies suggest that TERT may be an important target downstream of the PI3K/Akt signaling pathway to promote cell proliferation. However, whether PI3K/Akt also activates TERT and subsequently promotes vascular remodeling in PH remains to be explored.

Peroxisome-proliferator-activated receptor γ (PPAR-γ), a family member of nuclear receptors, has beneficial effects on cardiovascular systems that regulating adipogenesis and glucose metabolism [18]. Accumulating evidence have demonstrated that activation of PPAR-γ may be a novel PH therapeutic target. Pioglitazone, one of the most potent and selective synthetic agonists of PPAR-γ receptors, has been found to suppresses PASMCs proliferation and protect against PH development in experimental models [19,20]. Further studies have found that activation of PPAR-γ upregulates tumour suppressor gene PTEN and inhibit PI3K/Akt signaling pathway, thus to prevent pulmonary vascular remodeling in PH model [21,22]. Taken together, these findings lead to our hypothesis that PPAR-γ acts as a pivotal meadiator for PASMCs proliferation/migration and pulmonary vascular remodeling, these effects could be mediated by PI3K/Akt/c-MYC signaling pathway, and subsequently promotes TERT upregulation and pulmonary vascular remodeling. To address the above issues, we used mono-crotaline (MCT)-induced PH rat model to examine the role of PPAR-γ activation by pioglitazone on inhibition of TERT-mediated vascular remodeling in PH, thus to develop new insight and appropriate targets for the management of PH.

## 2 Materials and methods

### 2.1 Drugs and reagents

MCT (Must Bio-technology, Chengdu, China) was used to establish PH. BIBR1532 (APExBIO Techonologies, Houston, TX, USA) was used to impede the activity of telomerase. LY294002 (HY-MedChemExpress, Monmouth, NJ, USA). Pioglitazone (Cayman Chemical Company, Michigan, USA), was used to active PPAR-γ. Antibody against Akt, phospho-Akt (Ser473), c-Myc and GAPDH were purchased from Cell signaling Technology (Danvers, MA, USA). Polyclonal antibody against TERT were provided by Abcam (Cambridge, MA, USA).

### 2.2 Animals

Male Sprague-Dawley (SD) rats (190–200 g) were purchased from Xi'an Jiaotong University Animal Experiment Centre. All animal experiments were carried out in accordance with the Guide for Care and Use of Laboratory Animals of Xi'an Jiaotong University Animal Experiment Centre and approved by Laboratory Animal Care Committee of Xi'an Jiaotong University. They were kept under standard conditions with same light/dark cycle and housed in wire cages with free access to food and water.

### 2.3 Experimental design

The rats were randomly divided into five groups (n = 8 each group); control group, MCT group, MCT plus LY294002 treatment group, MCT plus BIBR1532 5 mg/kg treatment group and MCT plus BIBR1532 10 mg/kg treatment group, and MCT plus pioglitazone treatment group. Models of PH rats were established by a single intraperitoneal injection of MCT (60 mg/kg) on day 1 as previously described, pioglitazone (10 mg/kg) was given daily by gavage tube for 28 days. LY294002 (0.03 mg/kg) was given daily by intraperitoneal injection for 28 days. BIBR1532 were injected intraperitoneally with 5 mg/kg or 10 mg/kg twice a week for 28 days, respectively. Rats in control group were received an equal volume of 0.9% NaCl solution. The concentrations of the compounds were chosen based on previous studies [[23], [24], [25], [26]].

### 2.4 Measurement of RVSP and RVHI

Four weeks after MCT injection, all survived rats were anesthetized for hemodynamic measurements. As described previously, all rats underwent closed-chest right heart catheterization to detect right ventricle systolic pressure (RVSP), which was considered equal to pulmonary arterial pressure (PAP) [27]. After hemodynamic measurements, the harvested hearts were dissected into right ventricle RV, left ventricle LV plus interventricular septum, respectively. Each chamber was weighed separately to assess the right ventricular hypertrophy index (RVHI), a ratio of the weight of RV to LV plus S [RV/(LV + S)].

### 2.5 Histological analysis

Marginal right upper pulmonary lobes were fixed in 4% paraformaldehyde, and then the fixed lungs tissues were embedded in paraffin and sectioned at a thickness of 5 μm and stained with haematoxylin and eosin (HE) as previously reported [23]. Pulmonary vascular remodeling was evaluated by assessing the percentage of medial wall thickness (%WT). In each rat, ten pulmonary arteries (20–70 μm) were determined for structural integrity using a morphometric image system (Image J) [28]. The %WT was calculated as (external diameter-internal diameter)/external diameter × 100%.

### 2.6 Western blot analysis

Lung tissues from rats were lysed in RIPA Lysis Buffer, and then centrifuged at 13,000 rpm at 4 °C for 15 min. The supernatant was collected as sample proteins. Proteins were loaded and separated in SDS-PAGE gel and transferred to Polyvinylidene difluoride (PVDF) membranes as described previously [29]. After being blocked with 5% non-fat milk for 1 h at room temperature, membranes were incubated overnight at 4 °C with primary antibodies against TERT (1:1000 dilution, #ab191523, Abcam), p-Akt (1:1000 dilution, #13008, CST), t-Akt (1:1000 dilution, #4691, CST), c-Myc (1:1000 dilution, #5605, CST) and GAPDH (1:1000 dilution, TA802519, Origene) according to the manufacturer's instructions. Then the membranes were incubated with horseradish peroxidase conjugated with goat anti-rabbit or mouse IgG antibody. Immunoreactive bands were visualized with enhanced chemiluminescence (ECL) kit. Images were digitally captured using a ChemiDoc XPS System. The band densities were quantified using Quality One software (Bio-Rad).

### 2.7 Statistical analysis

All data were presented as mean ± SEM. One-way ANOVA followed by a Tukey post hoc test was conducted to analyse the differences between groups. *P* value < 0.05 was considered statistically significant.

## 3 Results

### 3.1 Effect of TERT inhibition on MCT-induced PH pulmonary vascular remodeling

**Fig. 1:**
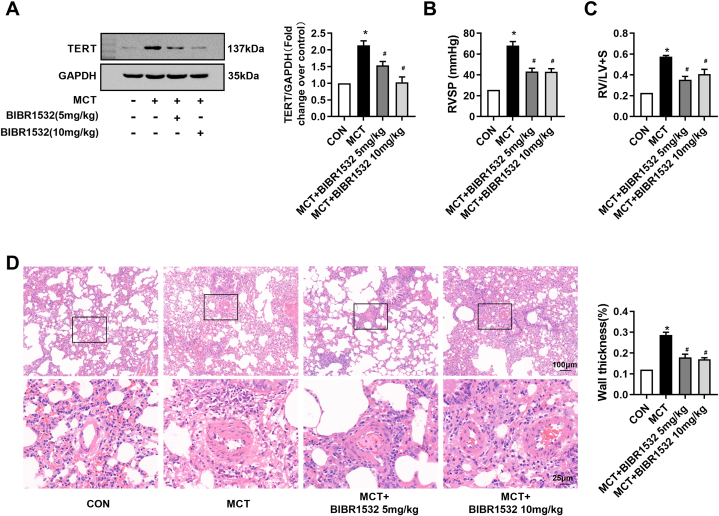
Effect of TERT inhibition on MCT-induced PH pulmonary vascular remodeling. (A) TERT protein level in lung tissues of rats was determined using immunoblotting, GAPDH served as a loading control (n = 4 each group). (B) Representative tracings of RVSP in each group (n = 6). (C) Changes of RV/LV + S in each group (n = 6). (D) HE-staining photomicrographs of small pulmonary vessels in different groups (n = 10 per rat). *P < 0.05 versus control group, #P < 0.05 versus MCT treated group.

To investigate whether TERT expression is elevated in lungs of MCT-induced rats, the protein level of TERT was detected by immunoblotting. As shown in Fig. 1A, the TERT expression was highly increased to (1.89 ± 0.28)-fold over control compared with the control group (*P* < 0.05). The result indicates that TERT is increased in MCT-treated PH rats.

Next, we examined whether suppression of TERT prevent MCT-induced PH development. BIBR1532, a potent and selective TERT inhibitor, was administrated to rat after MCT injection. As shown in Fig. 1A, administration of TERT inhibitor BIBR1532 of low (5 mg/kg) and high (10 mg/kg) dose significantly reduced the protein level of TERT (*P* < 0.05 vs. MCT-treated rats), indicating that TERT is effectively inhibited. In MCT-induced PH rats, RVSP significantly increased to 68.04 ± 7.92 mmHg versus 25.62 ± 8.11 mmHg in control rats (*P* < 0.05, Fig. 1B), suggesting that PH was successfully established in MCT-treated group. However, inhibition of TERT with BIBR1532 for low and high dose partially reduced RVSP to 43.2 ± 6.36 mmHg or 42.9 ± 6.15 mmHg (*P* < 0.05 vs. MCT group, Fig. 1B), respectively. Similar changes were found in RVHI. Fig. 1C showed that RVHI dramatically increased from 0.22 ± 0.06 in control rats to 0.57 ± 0.02 in MCT-treated rats (*P* < 0.05). After treatment with BIBR1532 of low and high dose, RVHI decreased to 0.34 ± 0.05 or 0.40 ± 0.07 (*P* < 0.05 vs. MCT group), respectively. These results suggest that inhibition of TERT prevents the development of MCT-induced PH.

Finally, we assessed the effects of TERT inhibition by BIBR1532 on MCT-induced pulmonary vascular remodeling using histological analysis. As depicted in Fig. 1D, the HE-staining results showed that medial wall thickness of small pulmonary arteries in MCT-treated rats were dramatically elevated compared with control rats. In addition, the quantitative morphometric analysis confirmed that %WT was significantly increased from (12.0 ± 1.0) % in control rats to (28.3 ± 1.5) % in MCT-treated rats (*P* < 0.05). However, after administration of BIBR1532 of low and high dose prevented MCT-induced increases in medial wall thickness, and reduced %WT to (17.9 ± 1.5) % or (17.0 ± 0.7) % (*P* < 0.05 vs. MCT group), respectively. Taken together, these results indicate that TERT inhibition dramatically prevents the pulmonary arterial remodeling by suppressing the proliferation of PASMCs.

### 3.2 PI3K/Akt/c-Myc pathway mediates TERT upregulation and pulmonary arterial remodeling in MCT-induced PH rats

**Fig. 2:**
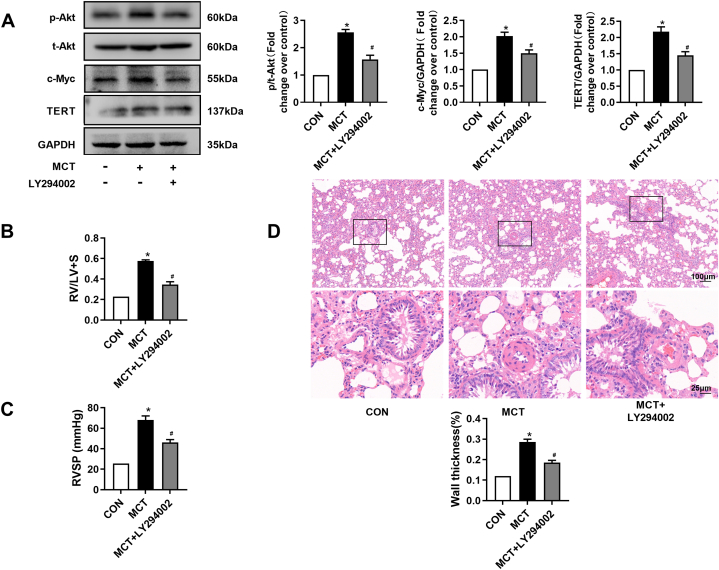
Inhibition of PI3K/Akt/c-Myc prevents MCT-induced PH in rats. (A) Protein level in lung tissues of rats was determined using immunoblotting, GAPDH served as a loading control (n = 4 each group. (B) Representative tracings of RVSP in each group (n = 6). (C) Changes of RV/LV + S in each group (n = 6). (D) HE-staining photomicrographs of small pulmonary vessels in different groups (n = 10 per rat). *P < 0.05 versus control group, #P < 0.05 versus MCT treated group.

It has been demonstrated that PI3K/Akt/c-Myc pathway regulates TERT expression in tumour cells. In this study, we investigated whether this above axis was involved in TERT expression in MCT-induced PH rats. As shown in Fig. 2A, administration of LY294002 suppressed MCT induced increases in p-Akt (1.57 ± 0.2 vs. 2.56 ± 0.10) and c-Myc (1.50 ± 0.10 vs. 2.02 ± 0.11). In addition, treatment with LY294002 significantly reduced MCT induced change in TERT expression from (2.18 ± 0.15)-fold to (1.45 ± 0.11)-fold, respectively (*P* < 0.05 vs. MCT group, Fig. 2A). Moreover, administration with LY294002 significantly reduced RVSP, RVHI, and wall thickness (*P* < 0.05 vs. MCT group, Fig. 2B–D). Together, these results suggest that PI3K/Akt/c-Myc signaling pathway mediates MCT-induced TERT upregulation and pulmonary arterial remodeling.

### 3.3 PPAR-γ activation by pioglitazone prevents the development of MCT-induced PH rats

**Fig. 3:**
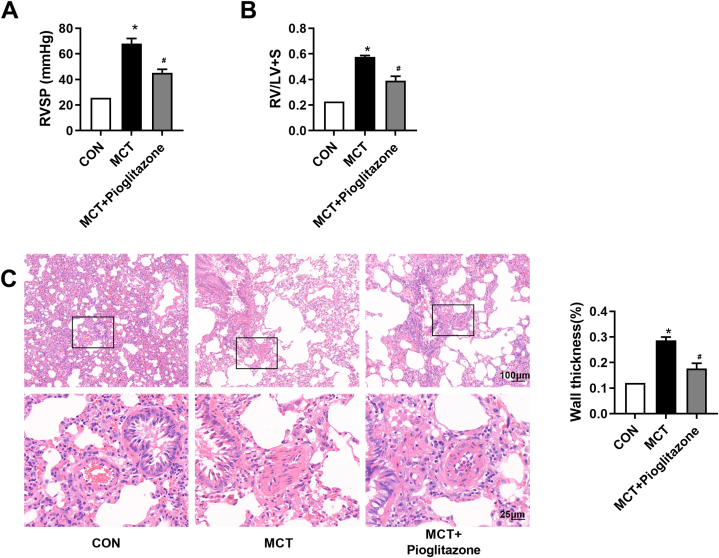
Pioglitazone prevents the development of MCT-induced PH in rats. (A) Representative tracings of RVSP in each group (n = 6). (B) Changes of RV/LV + S in each group (n = 6). (C) HE-staining photomicrographs of small pulmonary vessels in different groups (n = 10 per rat). *P < 0.05 versus control group, #P < 0.05 versus MCT treated group.

As shown in Fig. 3A and B, the RVSP and RVHI declined from 68.04 ± 7.92 mmHg and 0.57 ± 0.02 in MCT-treated rats to 45.2 ± 5.4 mmHg and 0.39 ± 0.06 in pioglitazone-treated PH rats, respectively (*P* < 0.05). Similar changes were observed in medial thickness of pulmonary arteries. Fig. 3C showed that medial thickness of pulmonary arteries was reduced. Quantitative morphometric analysis confirmed that %WT dropped to (17.7 ± 2.0) % in pioglitazone-treated PH rats (*P* < 0.05). These results suggest that activation of PPAR-γ by pioglitazone alleviates the pulmonary arterial remodeling by inhibiting PASMCs proliferation.

### 3.4 Mechanisms underlying PPAR-γ activation preventing MCT-induced PH rats

**Fig. 4:**
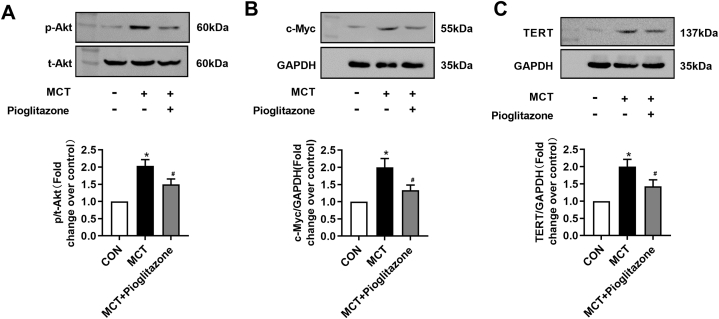
Mechanisms underlying Pioglitazone preventing MCT-induced PH rats. Expression levels of p/t-Akt (A), c-Myc (B), TERT(C) in lung tissues from different groups were determined using western blotting, GAPDH served as loading controls (n = 4 each group). *P < 0.05 versus control group, #P < 0.05 versus MCT treated group.

We further examined whether this beneficial effect of PPAR-γ activation is associated with the inhibition of Akt/c-Myc/TERT axis. Fig. 4A showed that the level of Akt phosphorylation increased to (2.03 ± 0.18)-fold compared with control group, while this level decreased to (1.5 ± 0.2)-fold over control in the pioglitazone-treated PH rats. Similarly, the level of c-Myc protein reached to (2.0 ± 0.2)-fold increase over control in MCT-treated rats and declined to (1.3 ± 0.1)-fold over control in the MCT plus pioglitazone-treated PH rats (Fig. 4B). In addition, Fig. 4C showed TERT protein level decreased to (1.4 ± 0.1)-fold over control in pioglitazone-treated PH rats (*P* < 0.05), suggesting that activation of PPAR-γ suppresses TERT expression by inhibiting Akt/c-Myc signaling pathway.

## 4 Discussion

In this study, we have found that TERT is highly expressed in MCT-induced PH rats and inhibition of TERT expression significantly prevents MCT-induced PH progression by suppressing pulmonary vascular remodeling. In addition, activation of PPAR-γ might be associated with suppression of Akt/c-Myc pathway and subsequently inhibition of TERT expression, thus to alleviate PH development. Our study provides a new molecular basis whereby inhibition of TERT function might benefit for PH management by preventing pulmonary vascular remodeling.

Telomerase activity constitutes the addition of noncoding TTAGGG nucleotide repeats, preventing the ends of chromosomes from deteriorating [30]. TERT, an important component of telomerase, maintains telomere homeostasis by lengthening telomeric DNA [31]. It has been confirmed that telomerase activation is an early event in the development of cancers, especially the TERT, which plays an important role in this process [32,33]. TERT is expressed at low levels or not detectable in normal somatic cells and tissues while its expression is upregulated in most carcinoma cells and highly proliferative organs [34]. Recently, several studies suggested TERT expression or activity increase in lungs from patients with idiopathic PH and animal models with PH. These is consistent with the results of our current study, which showed that overexpression of TERT was found in MCT-induced PH rats, which once again verified the reliability of our in vitro experiment [7,8,12,35]. To further investigate the effects of TERT in PH, we used BIBR1532 as a pharmacological inhibitor. The inhibition of TERT resulted in reduced wall thickness, RVSP and right hypertrophy. These results constitute the causal role of TERT in pulmonary vascular remodeling in PH.

PI3K/Akt signaling pathway participates in cell proliferation, differentiation, apoptosis, glucose transport and other cellular functions. A variety of growth factors, such as platelet-derived growth factor (PDGF) and hypoxia, activates PI3K/Akt signaling pathway, promoting proliferation and migration of various tumour cells. Recent studies have demonstrated that inhibition of PI3K/Akt pathway inhibits TERT expressions, which further diminishes proliferation of lung adenocarcinoma cells and breast cancer cells [36,37]. These studies suggest that TERT is an important target gene which downstream the PI3K/Akt signaling pathway and promote cell proliferation. In HaCaT cells, chromatin immunoprecipitation experiments have shown that c-MYC regulates the transcriptional activity of TERT by enhanced binding to TERT promoters [38]. In present study, we found that inhibition of PI3K/Akt pathway significantly suppressed c-Myc and TERT upregulation and pulmonary arterial remodeling in MCT induced PH rats model.

Peroxisome proliferator-activated receptor γ (PPAR-γ) is a member of the nuclear receptor family. PPAR-γ forms a heterodimer with the retinoic acid X receptor family and binds to DNA containing The repeated 5′-AGGTCA-3′ sequence of PPAR response elements (PPAR response elements, PPREs) regulate downstream target genes and signal pathways, mediate fat metabolism, cell proliferation, differentiation and migration and other physiological processes, which are considered to be one An important endogenous protective molecule [39]. Clinically, PPAR-γ agonists such as pioglitazone have been used to treat type 2 diabetes [40]. In recent years, studies have further suggested that activation of PPAR-γ may have potential clinical application value in a variety of diseases other than diabetes such as cardiovascular diseases, tumours, and inflammation [19]. Another study has indicated that PPAR-γ down-regulates the expression of TERT, depending on Wnt/β-Catenin signal way in GC cell lines [38]. In this study, we found that activation of PPAR-γ by pioglitazone suppressed Akt and c-Myc expressions, which further downregulated TERT, thus to prevent pulmonary vascular remodeling in PH. Our study offers a novel perspective of the mechanisms responsible for the protective effects of PPAR-γ activation on experimental PH.

## 5 Conclusion

In summary, we determine that TERT upregulation significantly promotes the development of MCT-induced PH, and activation of PPAR-γ suppresses pulmonary vascular remodeling by inhibition of Akt/c-Myc/TERT axis in MCT-induced PH. Targeting TERT signaling pathways may have potential value in therapeutic interventions for PAH.

## Ethics approval and consent to participate

This article does not contain any studies with human participants or animals performed by any of the authors.

## Author contribution statement

Tafseel Hussain, Limin Chai, Yan Wang, Qianqian Zhang, Jian Wang, Wenhua Shi, Qingting Wang, Manxiang Li, Xinming Xie: Conceived and designed the experiments; Performed the experiments; Analyzed and interpreted the data; Contributed reagents, materials, analysis tools or data; Wrote the paper.

## Funding statement

Xinming Xie was supported by 10.13039/501100001809National Natural Science Foundation of China [81800052].

## Consent for publication

All authors have seen the manuscript and approved to submit to your journal.

## Data availability statement

Data included in article/supp. material/referenced in article.

## Declaration of interest's statement

The authors declare that they have no known competing financial interests or personal relationships that could have appeared to influence the work reported in this paper.
